# Beat Frequency Estimation in a Square He–Ne Ring Laser Gyroscope: Joint I/Q Channel Calibration and Kalman Filtering

**DOI:** 10.3390/s26175590

**Published:** 2026-09-03

**Authors:** Lei Shi, Zhifu Luo, Jing Hu, Jiajia Lu, Dingbo Chen, Zilong Xie, Suyong Wu, Zhongqi Tan

**Affiliations:** College of Advanced Interdisciplinary Studies, National University of Defense Technology, Changsha 410073, China; shileinudt@sina.com (L.S.); 15616218757@163.com (J.H.); jieya18229786113@163.com (J.L.); chendingbo15@nudt.edu.cn (D.C.); xiezilong0731@foxmail.com (Z.X.); sywu2001@163.com (S.W.)

**Keywords:** ring laser gyroscope, multichannel fusion, in-phase/quadrature demodulation, generalized least squares, extended Kalman filter, unscented Kalman filter, innovation diagnostics

## Abstract

A gain–phase-calibrated four-channel in-phase/quadrature (I/Q) fusion and recursive beat-frequency estimation framework is developed for a square He–Ne ring laser gyroscope. Calibration-derived channel parameters enable ordinary least-squares (OLS) fusion, while residual covariance estimation provides a generalized least-squares (GLS) extension. Known-ground-truth simulations verify the theoretical fusion gain, covariance propagation, and consistency of the recursive estimation framework. A chronologically partitioned experimental record is used for calibration, parameter tuning, and held-out validation. The proposed fusion improves spectral signal-to-noise ratio compared with individual channels, and the independently tuned extended and unscented Kalman filters achieve millihertz-level frequency estimation accuracy. Innovation analysis further reveals remaining carrier-synchronous temporal correlations, indicating the importance of more complete residual modeling for future stochastic refinement.

## 1. Introduction

Ring laser gyroscopes provide continuous measurements of rotation through the Sagnac beat frequency and have been used in high-resolution Earth-rotation monitoring, geophysical observation, and tests of fundamental physics [1,2,3,4,5,6]. Their achievable readout stability depends not only on the optical cavity and laser dynamics but also on active-medium evolution, backscatter-related nonlinearities, acquisition electronics, and the subsequent extraction of the beat signal. Recent studies have examined non-reciprocal frequency contributions from the active medium and laser dynamics [7,8], as well as hysteresis of the lock-in range in He–Ne ring laser gyroscopes [9]. In the recorded multichannel signals considered here, the readout is additionally affected by channel-dependent offsets, gain mismatch, effective phase errors, and correlated residuals.

The present work does not introduce an active-medium, backscatter, or lock-in correction model; instead, it addresses calibrated multichannel beat-note fusion and recursive frequency estimation. Multichannel detection provides redundant observations of the same beat signal and can therefore improve readout robustness. However, direct averaging does not account for the phase geometry of the detector channels and does not explicitly recover a normalized quadrature pair. Ordinary least-squares (OLS) estimation provides a natural framework for reconstructing common orthogonal components, while generalized least squares (GLS) can incorporate unequal and correlated channel residuals [10,11]. The practical benefit depends on the accuracy of the gain–phase calibration and residual-covariance model.

After quadrature recovery, instantaneous frequency can be estimated using analytic-signal phase differentiation or finite-record spectral methods [12,13]. The EKF and UKF are standard nonlinear state-space estimators [14,15,16]. In the present work, they are configured to jointly track phase, frequency, amplitude, and quadrature offsets. Their performance depends strongly on the assumed process and measurement covariances [16,17]. Consistency-based auto-tuning methods likewise show that covariance selection can be sensitive to parameter ambiguity and the chosen statistical criterion [18]. Moreover, tuning and evaluating the filters on the same record can lead to optimistic performance estimates. A rigorous assessment therefore requires both chronologically separated experimental data and known-ground-truth simulations that distinguish implementation correctness from model mismatch.

Accordingly, the study tested whether (i) Calibration-frozen joint gain–phase fusion improves the held-out spectral signal-to-noise ratio (SNR) relative to the mean single-channel baseline, (ii) independently tuned recursive estimators remain numerically stable on chronologically held-out data, and (iii) near-reference marginal innovation statistics can coexist with substantial temporal dependence.

To address these limitations, we develop a processing chain that combines joint gain–phase-calibrated four-channel in-phase/quadrature (I/Q) fusion with independently tuned recursive beat-frequency estimation. This work makes four contributions. First, joint gain–phase-calibrated OLS and covariance-aware GLS fusion are formulated with explicit residual-covariance propagation. A controlled fixed-seed simulation verifies the ideal fusion gain and amplitude-preserving reconstruction, while separate known-ground-truth Monte Carlo experiments verify covariance propagation under nonideal channel geometry and correlated residuals. Second, the experimental record is divided chronologically into Calibration, Tuning, and held-out Validation segments, ensuring that neither fusion calibration nor filter selection uses Validation data. Third, the EKF and UKF are tuned independently, and two process-noise discretizations are compared: the historical empirical covariance and an integrated phase–frequency random-walk covariance. Fourth, marginal innovation consistency is distinguished from temporal whiteness through autocorrelation, spectral, phase-conditioned, and post hoc residual-model diagnostics, with Validation-informed analyses explicitly separated from the formal estimator. This distinction prevents near-nominal mean normalized innovation squared (NIS) values from being interpreted as proof of a complete stochastic measurement model.

Detailed derivations, implementation settings, and full diagnostic tables are retained in Appendix A, while the principal simulation and experimental results are reported in the main text.

## 2. Materials and Methods

### 2.1. Experimental System, Data Partition, and Frozen Calibration

A self-developed square He–Ne ring laser gyroscope with 400-mm side length and 632.8 nm nominal wavelength was observed using four selected elements of a 16-element avalanche photodiode (APD) array (First Sensor AG, Berlin, Germany) [19]. The detector voltages were recorded at a nominal 1 kHz per channel using a multiplexed NI USB-6009 acquisition device (National Instruments Corporation, Austin, TX, USA) [20]. The available record did not preserve the interchannel conversion timing or complete analog-front-end settings. The analyzed record contained 150,802 finite samples over 150.801 s.

The record was partitioned chronologically into Calibration (0–20 s), Tuning (20–80 s), and held-out Validation (80–150.801 s). Calibration estimated the channel offsets, relative gains, phase offsets, initial beat frequency, and channel residual covariance. Tuning selected the frequency-random-walk amplitude $\sigma_{f,\mathrm{rw}}$$\left(\mathrm{Hz}\;s^{-1/2}\right)$, which controls the frequency-process-noise strength, and the dimensionless measurement-covariance scale factor $R_{\mathrm{scale}}$, which scales the Calibration-derived OLS residual covariance term, separately for each filter and process model. All parameters were frozen before Validation.

At the Calibration-derived beat-frequency estimate $f_{\mathrm{cal}}$, each offset-corrected channel *k* was fitted by sine, cosine, and constant terms, where $a_{k}$ and $c_{k}$ denote the fitted sine and cosine coefficients, respectively. The normalized relative gain ${\hat{g}}_{k,\mathrm{cal}}$ and the channel-1-referenced phase offset ${\hat{\varphi }}_{k,\mathrm{cal}}$ were

(1) $${\hat{g}}_{k,\mathrm{cal}}=\frac{\sqrt{a_{k}^{2}+c_{k}^{2}}}{\frac{1}{4}\sum_{j=1}^{4}\sqrt{a_{j}^{2}+c_{j}^{2}}},\;{\hat{\varphi }}_{k,\mathrm{cal}}=\mathrm{atan}2\left(c_{k},a_{k}\right)-{\hat{\varphi }}_{1,\mathrm{cal}}.$$

**Figure 1:**
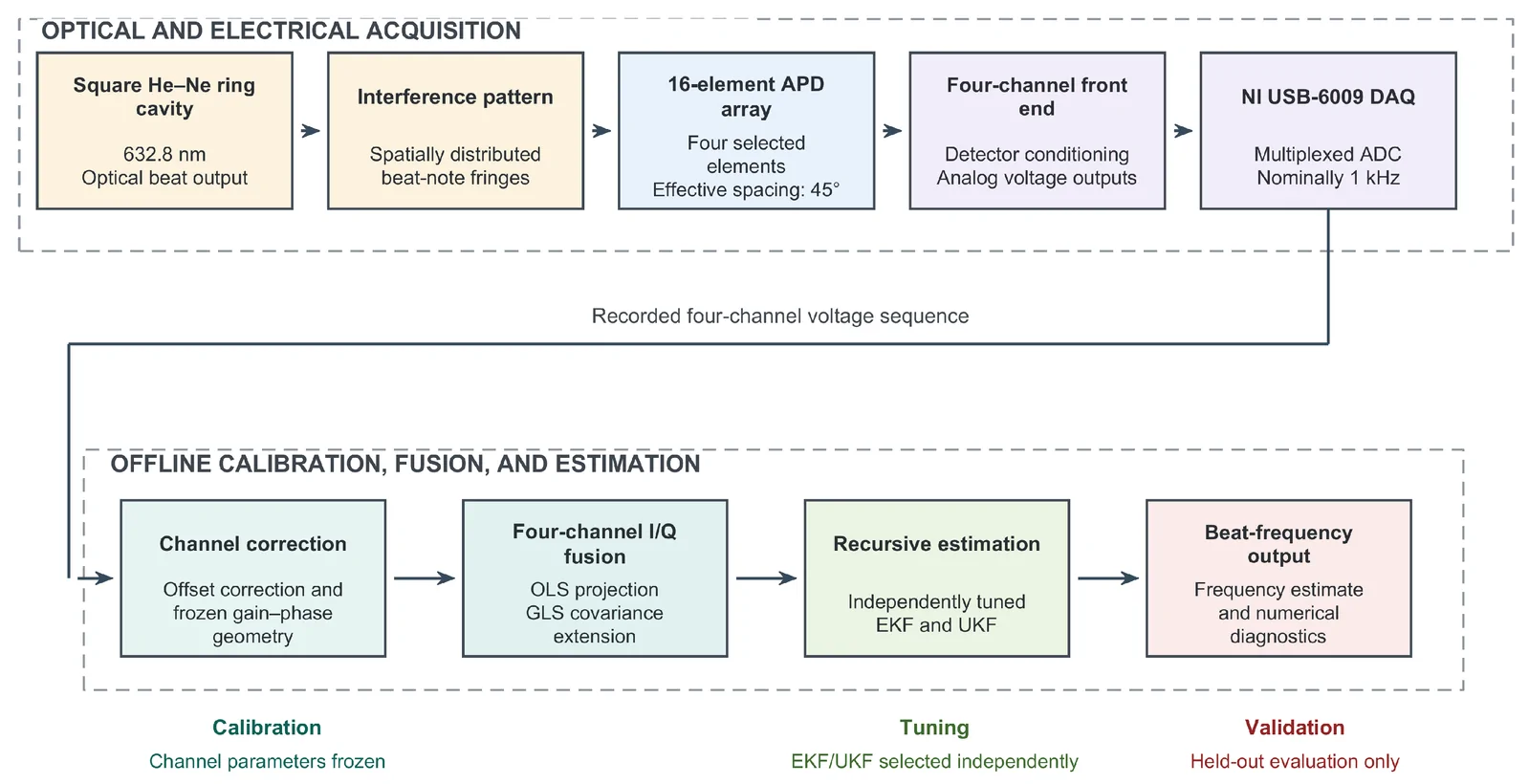
Experimental acquisition and offline processing chain. Calibration and Tuning were completed before the held-out Validation segment was evaluated. APD, avalanche photodiode; DAQ, data acquisition; I/Q, in-phase/quadrature; OLS, ordinary least squares; GLS, generalized least squares; EKF, extended Kalman filter; UKF, unscented Kalman filter.

The covariance of the four fixed-frequency regression residuals defined ${\hat{\Sigma }}_{\mathrm{ch},\mathrm{cal}}$, the Calibration-derived four-channel residual covariance matrix, and the frozen GLS matrix. Because these residuals can include waveform and short-term signal mismatch, this matrix is interpreted as a residual covariance rather than pure detector-noise covariance. The experimental acquisition and offline processing chain is summarized in Figure 1.

### 2.2. Gain–Phase Calibration and Four-Channel I/Q Fusion

After subtracting channel offsets estimated from Calibration, the four detector outputs were represented as

(2) $$s_{n}=Hu_{n}+n_{n},\;u_{n}={\left[\begin{array}{cc} I_{n} & Q_{n} \end{array}\right]}^{T},$$

where $s_{n}\in R^{4}$ is the offset-corrected four-channel detector vector, $u_{n}$ is the underlying I/Q component vector, $H\in R^{4\times 2}$ is the Calibration-frozen gain–phase mixing matrix, and $n_{n}\in R^{4}$ is the residual vector. The quadrature components are $I_{n}=A_{n}\sin\theta_{n}$ and $Q_{n}=A_{n}\cos\theta_{n}$, where $A_{n}$ and $\theta_{n}$ denote the beat-signal amplitude and phase, respectively.

(3) $$H_{k,:}={\hat{g}}_{k,\mathrm{cal}}\left[\begin{array}{cc} \cos{\hat{\varphi }}_{k,\mathrm{cal}} & \sin{\hat{\varphi }}_{k,\mathrm{cal}} \end{array}\right].$$

The offsets, relative gains, and phase offsets were estimated using only Calibration and were frozen before Tuning and Validation. The residual vector had covariance $\Sigma_{\mathrm{ch}}$, which was estimated from Calibration regression residuals.

For full-column-rank H, the ordinary least squares (OLS) and generalized least squares (GLS) fusion matrices were

(4) $$W_{\mathrm{OLS}}={\left(H^{T}H\right)}^{-1}H^{T},\;W_{\mathrm{GLS}}={\left(H^{T}\Sigma_{\mathrm{ch}}^{-1}H\right)}^{-1}H^{T}\Sigma_{\mathrm{ch}}^{-1}.$$

The fused components were ${\hat{u}}_{m,n}=W_{m}s_{n}$ for m∈{OLS,GLS}. Their propagated residual covariances were

(5) $$R_{\mathrm{OLS}}=W_{\mathrm{OLS}}\Sigma_{\mathrm{ch}}W_{\mathrm{OLS}}^{T},\;R_{\mathrm{GLS}}={\left(H^{T}\Sigma_{\mathrm{ch}}^{-1}H\right)}^{-1}.$$

OLS was used as the primary recursive-filter input. GLS was evaluated as a covariance-aware extension because the estimated residual covariance can include detector noise, waveform mismatch, and short-term signal variation.

For unit gains, ideal phase offsets ${\left[0,\pi /4,\pi /2,3\pi /4\right]}^{T}$, and independent equal-variance channel residuals, $H^{T}H=2I_{2}$ and

(6) $$R_{\mathrm{OLS}}=\frac{\sigma^{2}}{2}I_{2},\;G_{\mathrm{ideal},\mathrm{dB}}=10\log_{10}\left(2\right)=3.0103\;\mathrm{dB}.$$

This value is an ideal equal-noise reference rather than a universal upper bound for the experimental unequal-noise case. The full OLS/GLS derivations and the ideal equal-noise calculation are given in Appendix A.1.

### 2.3. EKF and UKF Beat-Frequency Estimation

The fused OLS I/Q sequence was processed by the extended Kalman filter (EKF) and unscented Kalman filter (UKF) estimators using the common state

(7) $$x_{k}={\left[\begin{array}{ccccc} \theta_{k} & \omega_{k} & A_{k} & b_{I,k} & b_{Q,k} \end{array}\right]}^{T},\;\theta_{k+1}=\theta_{k}+\omega_{k}T_{s}.$$

The sample index *n* is used for the fusion stage (Section 2.2 and Appendix A.1), whereas *k* indexes the recursive-filter stage; both refer to the same 1 kHz sample grid.

Here, $\theta_{k}$, $\omega_{k}$, and $A_{k}$ denote the beat phase, angular frequency, and amplitude, respectively; $b_{I,k}$ and $b_{Q,k}$ represent the estimated bias terms of the fused I/Q components, and $T_{s}$ is the sampling interval.

The measurement model was

(8) $$z_{k}=\left[\begin{array}{c} A_{k}\sin\theta_{k}+b_{I,k}\\ A_{k}\cos\theta_{k}+b_{Q,k} \end{array}\right]+v_{k}.$$

Two process covariances were compared. The empirical model used independent state increments, whereas the integrated model additionally retained the phase variance and phase–frequency covariance generated by integrating frequency random walk. With $q_{\omega }={\left(2\pi \sigma_{f,\mathrm{rw}}\right)}^{2}T_{s}$, where $q_{\omega }$ denotes the per-sample angular-frequency process-noise variance, their phase–frequency blocks were

(9) $$Q_{\mathrm{emp}}^{\left(\theta ,\omega \right)}=\left[\begin{array}{cc} 10^{-10} & 0\\ 0 & q_{\omega } \end{array}\right],\;Q_{\mathrm{int}}^{\left(\theta ,\omega \right)}=\left[\begin{array}{cc} 10^{-10}+q_{\omega }T_{s}^{2}/3 & q_{\omega }T_{s}/2\\ q_{\omega }T_{s}/2 & q_{\omega } \end{array}\right].$$

The amplitude and I/Q bias variances were fixed at $10^{-10}$, $10^{-12}$, and $10^{-12}$ per sample, respectively. The Calibration-frozen OLS residual covariance defined

(10) $$R=R_{\mathrm{scale}}W_{\mathrm{OLS}}{\hat{\Sigma }}_{\mathrm{ch},\mathrm{cal}}W_{\mathrm{OLS}}^{T}+10^{-12}I_{2}.$$

The filters used a common input-dependent initialization based on the first 999 fused samples of each recursive run and the beat frequency frozen from Calibration. The count of 999 arose from the archived operation $\left\lfloor f_{s}\right\rfloor$ on a double-precision sampling-rate value slightly below the nominal 1 kHz. Both estimators used the same 18–22 Hz frequency limits. The EKF used a Joseph covariance update; the UKF used α=0.5, β=2, and κ=0, circular phase averaging, and 11 sigma points. Complete initialization, recursion, constraint handling, and numerical-audit details are provided in Appendix A.2.

For innovation $\nu_{k}$ and predicted covariance $S_{k}$, the normalized innovation squared was

(11) $$\epsilon_{k}=\nu_{k}^{T}S_{k}^{-1}\nu_{k}.$$

For the two-dimensional innovation, the marginal NIS reference has mean 2, with one-sided 95% and 99% thresholds of 5.9915 and 9.2103 [21]. Temporal adequacy was assessed using autocorrelation and Ljung–Box tests [22] and Welch spectra [23]. Cholesky-normalized innovations and phase-conditioned means were additionally examined as study-specific diagnostics. These diagnostics were evaluated only after all formal parameters had been frozen.

### 2.4. Independent Tuning and Held-Out Evaluation

A common filter-independent Tuning reference was formed from covariance-weighted sliding-window fast Fourier transform (FFT) estimates of the four offset-corrected detector channels. Each frequency estimate used a 300-sample symmetric Hann window, 10-sample step, 16,384-point transform, and 15–25 Hz search band. This common reference was used for all EKF/UKF and process-model scans.

Each of the four tasks evaluated 40 logarithmically spaced $\sigma_{f,\mathrm{rw}}$ values from $10^{-15}$ to $10^{-2}$ and 20 $R_{\mathrm{scale}}$ values from $10^{-3}$ to $10^{2}$. Selection used a predefined composite of mean and median normalized innovation squared (NIS) agreement, frequency dispersion, peak-to-peak range, and root-mean-square error (RMSE) relative to the common Tuning trend. Candidates were required to be numerically valid and were preferentially selected from $1.5\leq \bar{\epsilon }\leq 2.5$ and $1.1\leq \tilde{\epsilon }\leq 1.8$. The full composite cost, selected-parameter table, near-optimal plateaus, and numerical audit are provided in Appendix A.5.

Primary fusion performance was measured over complete Validation using the MATLAB R2024b Signal Processing Toolbox implementation snr(x,fs) with the same effective sampling frequency and sample-mean removal for all channels and fused components. Fusion gains were referenced to the arithmetic mean of the four single-channel SNR values in decibels. Frequency and innovation statistics excluded the first and last 1 s of Validation, leaving 68,802 samples. Bias and RMSE were referenced to a scalar filter-independent pseudo-reference frozen from the Tuning-only sliding-window FFT trend and therefore do not represent absolute truth-referenced accuracy.

All simulations, recursive-filter scans, statistical analyses, and figures were generated using MATLAB version 24.2 (R2024b; The MathWorks, Inc., Natick, MA, USA). The Signal Processing Toolbox version 24.2 (R2024b) was used for the SNR evaluation.

### 2.5. Known-Ground-Truth Simulations

Four complementary simulation checks were used. First, a reproducible 100 s ideal four-channel record at 1 kHz used unit gains, ideal $45^{{}^{\circ}}$ spacing, amplitude 1.5, channel-noise standard deviation 0.2, and MATLAB Mersenne Twister seed 20250308. It verified the 3.0103 dB equal-noise OLS reference, amplitude preservation, and analytical covariance propagation.

Second, a nonideal gain–phase geometry with correlated residuals was used to verify OLS/GLS covariance propagation. A total of 500,000 Gaussian residual vectors were generated using seed 21260300 and propagated through the fixed fusion matrices.

Third, matched-model recursive-filter consistency was evaluated over 100 paired 60 s realizations at 1 kHz. The Tracking EKF and UKF used the same simulated truth and measurement record within each realization. The recursive-filter simulations adopted the same angular-frequency state convention as the experimental estimators, with the frequency state represented as angular frequency ω and the UKF scaling parameters fixed to α=0.5, β=2, and κ=0. The corresponding angular-frequency process noise was generated using the same 2π conversion from $\sigma_{f,\mathrm{rw}}$ as the experimental formulation. Mersenne Twister seeds ranged from 20310302 to 20310401, and the first and last 1 s were excluded from the consistency statistics. The theoretical mean references were 2 for NIS, 1 for frequency-state NEES, and 5 for full-state NEES. Detailed simulation matrices and seed mappings are provided in Appendix A.3.

Fourth, dynamic-response behavior was examined using constant, step, ramp, and sinusoidal frequency truths. Each truth profile was evaluated over 100 paired Monte Carlo measurement records for predefined low-process and Tracking configurations of both filters. The dynamic-response simulations used the same angular-frequency state convention and UKF scaling parameters as the matched-model simulations. The dynamic study therefore comprised 400 generated records and 1600 filter executions. The reported RMSEs are ensemble means with 95% confidence intervals; complete profile definitions, seed mappings, and representative trajectories are provided in Appendix A.4.

### 2.6. Covariance-Transfer and Residual Diagnostics

A fitted-sinusoid transfer diagnostic tested whether the channel residual covariance frozen from Calibration quantitatively predicted held-out fused-component SNR gains. Signal powers were fitted on the evaluated Validation interval, whereas the channel covariance and fusion matrices remained frozen from Calibration; the analysis was therefore a conditional covariance-transfer diagnostic rather than a prospective SNR prediction. The complete definitions are given in Appendix A.6.

After the formal EKF and UKF parameters had been frozen, Cholesky-normalized innovations were examined using sample autocorrelation, Ljung–Box tests, Welch auto- and cross-spectra, carrier-harmonic peak extraction, magnitude-squared I/Q coherence, and phase-conditioned means. A Tuning-fitted phase-harmonic model and a subsequent vector autoregressive (VAR) model were evaluated only as Validation-informed post hoc diagnostics. Neither model was used to select or modify a formal estimator. Full definitions and the complete harmonic table are provided in Appendix A.7.

## 3. Results

### 3.1. Experimental Calibration and Four-Channel Fusion

Calibration produced relative gains ${\left[0.8014,\;1.2741,\;0.8394,\;1.0851\right]}^{T}$ and phase offsets ${\left[0,\;46.910,\;91.104,\;134.930\right]}^{{}^{\circ}}$. The resulting gain–phase design matrix had rank 2 and condition number 1.115. Relative to OLS, GLS reduced the Calibration-propagated I- and Q-component residual variances by 0.298% and 3.358%, corresponding to variance-based improvements of 0.013 and 0.148 dB. Independent reconstruction reproduced the frozen OLS weights at stored precision and the GLS weights to within $2.78\times 10^{-16}$.

The held-out OLS I/Q spectral-SNR gains were 2.247 and 2.373 dB, increasing to 2.450 and 2.814 dB with GLS. A separate fitted-sinusoid transfer diagnostic reproduced the frozen fusion histories and propagated covariances at machine precision, but the Calibration residual covariance underpredicted the held-out fitted-sinusoid gains by 2.287–3.081 dB. The discrepancy persisted in 1 s and 5 s windows, indicating incomplete transfer of the Calibration residual statistics rather than an algebraic fusion error. The corresponding covariance-transfer analysis is reported separately in Section 3.4. The complete held-out Validation SNR values and the corresponding OLS and GLS fusion gains are summarized in Table 1.

### 3.2. Independent Tuning and Held-Out Frequency Estimation

All four 800-candidate scans selected numerically valid candidates from the strict marginal-NIS region. The EKF and UKF independently selected the same measurement-covariance scale, $R_{\mathrm{scale}}=0.0695$, but different low $\sigma_{f,\mathrm{rw}}$ grid points. Both selected points lay on broad near-optimal plateaus, so they should not be interpreted as uniquely identified physical random-walk strengths. The empirical and integrated process models produced identical selected metrics and Validation trajectories at stored precision. The selected values were $\sigma_{f,\mathrm{rw}}=1.0000\times 10^{-11}$ for both EKF process models and $4.642\times 10^{-12}$ for both UKF process models, with $R_{\mathrm{scale}}=0.0695$ in all four tasks. The full selected-parameter table, near-optimal plateau ranges, and numerical audit are provided in Appendix A.5. The corresponding held-out Validation performance metrics for the independently tuned EKF and UKF are summarized in Table 2.

On this single trimmed Validation record, the EKF had lower frequency dispersion, peak-to-peak range, and pseudo-reference-relative RMSE than the UKF. These differences describe within-record repeatability and do not establish absolute accuracy or general superiority. Neither filter experienced a frequency-limit activation, innovation-covariance Cholesky failure, or adaptive regularization event within the trimmed Validation interval.

**Figure 2:**
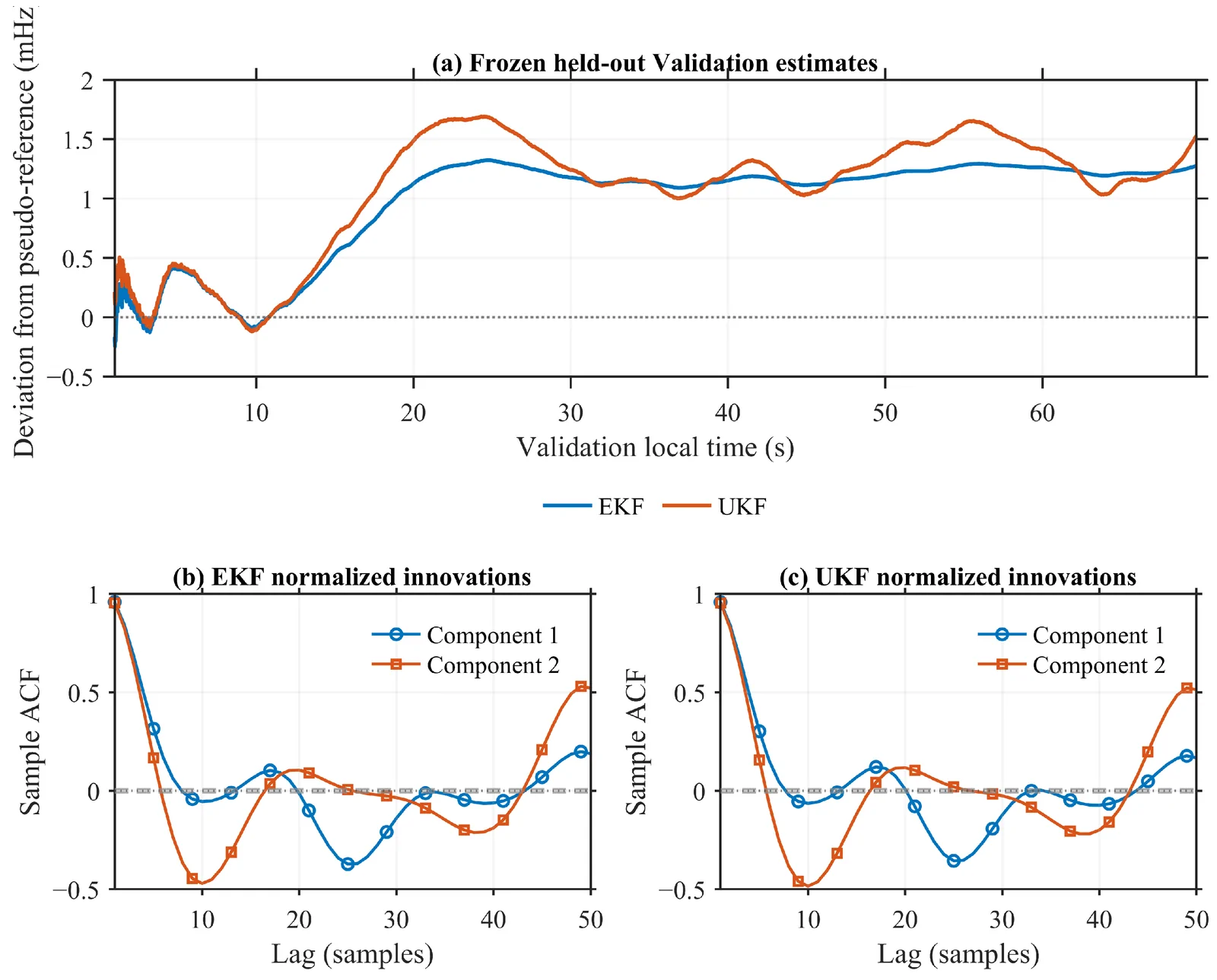
Held-out Validation frequency-estimation and innovation diagnostics. (**a**) EKF and UKF frequency deviations from the scalar pseudo-reference frozen from the Tuning-only sliding-window FFT trend. (**b**,**c**) Positive-lag autocorrelations of the two Cholesky-normalized innovation components for the EKF and UKF, respectively. Dashed horizontal lines denote the nominal pointwise white-noise bounds. The displayed frequency deviations are relative to a frozen pseudo-reference and are not absolute truth-referenced errors. ACF, autocorrelation function; EKF, extended Kalman filter; UKF, unscented Kalman filter.

Figure 2 summarizes the frozen Validation frequency deviations and the principal temporal innovation diagnostics.

### 3.3. Known-Ground-Truth Simulation Verification

The fixed-seed ideal four-channel record yielded I- and Q-component OLS gains of 3.0178 and 3.0071 dB, respectively, compared with the 3.0103 dB theoretical equal-noise reference. The maximum absolute noise-free I/Q reconstruction error was $2.22\times 10^{-16}$, and the relative Frobenius error between the empirical and analytically propagated output covariances was 0.2391%. Under the separate nonideal gain–phase geometry with correlated channel residuals, the corresponding covariance errors were 0.160% for OLS and 0.139% for GLS.

In the 100-realization matched-model experiment, the mean NIS values were 1.9988 for both the EKF and UKF, compared with the two-dimensional theoretical reference of 2. The mean frequency-state normalized estimation error squared (NEES) values were 1.0163 and 1.0164 for the EKF and UKF, respectively, while the mean full-state NEES values were 5.0258 and 4.9900, compared with theoretical references of 1 and 5, respectively. The full-state central-95% coverage rates were 94.488% for the EKF and 94.553% for the UKF. Taken together, these simulations verify the fusion, covariance-propagation, NIS, and NEES implementations under their correctly specified simulation models. The complete paired consistency statistics are listed in Table 3.

The dynamic-response study exposed the expected trade-off between steady-state smoothness and responsiveness. The low-process configurations achieved the smallest constant-frequency RMSEs but lagged strongly under step, ramp, and sinusoidal truths, whereas the Tracking configurations reduced the dynamic errors. The ensemble RMSE results are summarized in Table 4; representative trajectories are provided in Appendix A.4. For the sinusoidal truth profile, the Tracking EKF and UKF preserved approximately unity modulation amplitude and exhibited a phase delay of approximately 0.468 s, while the low-process configurations showed stronger attenuation and larger delay. These results characterize the algorithm-level dynamic response of the recursive estimators rather than the complete bandwidth of the optical and acquisition hardware.

### 3.4. Calibration-to-Validation Covariance Transfer

The common fitted beat frequency over trimmed Validation was 20.283850 Hz. The reconstructed OLS and GLS sequences agreed with the frozen Stage-2 histories to within $1.11\times 10^{-16}$ in maximum absolute difference. The full-interval measured gains were 4.581, 3.659, 4.648, and 4.347 dB for OLS I, OLS Q, GLS I, and GLS Q, respectively, whereas the corresponding Calibration-covariance predictions were 1.554, 1.371, 1.567, and 1.518 dB. The measured-minus-predicted discrepancies were therefore 3.027, 2.287, 3.081, and 2.829 dB.

**Figure 3:**
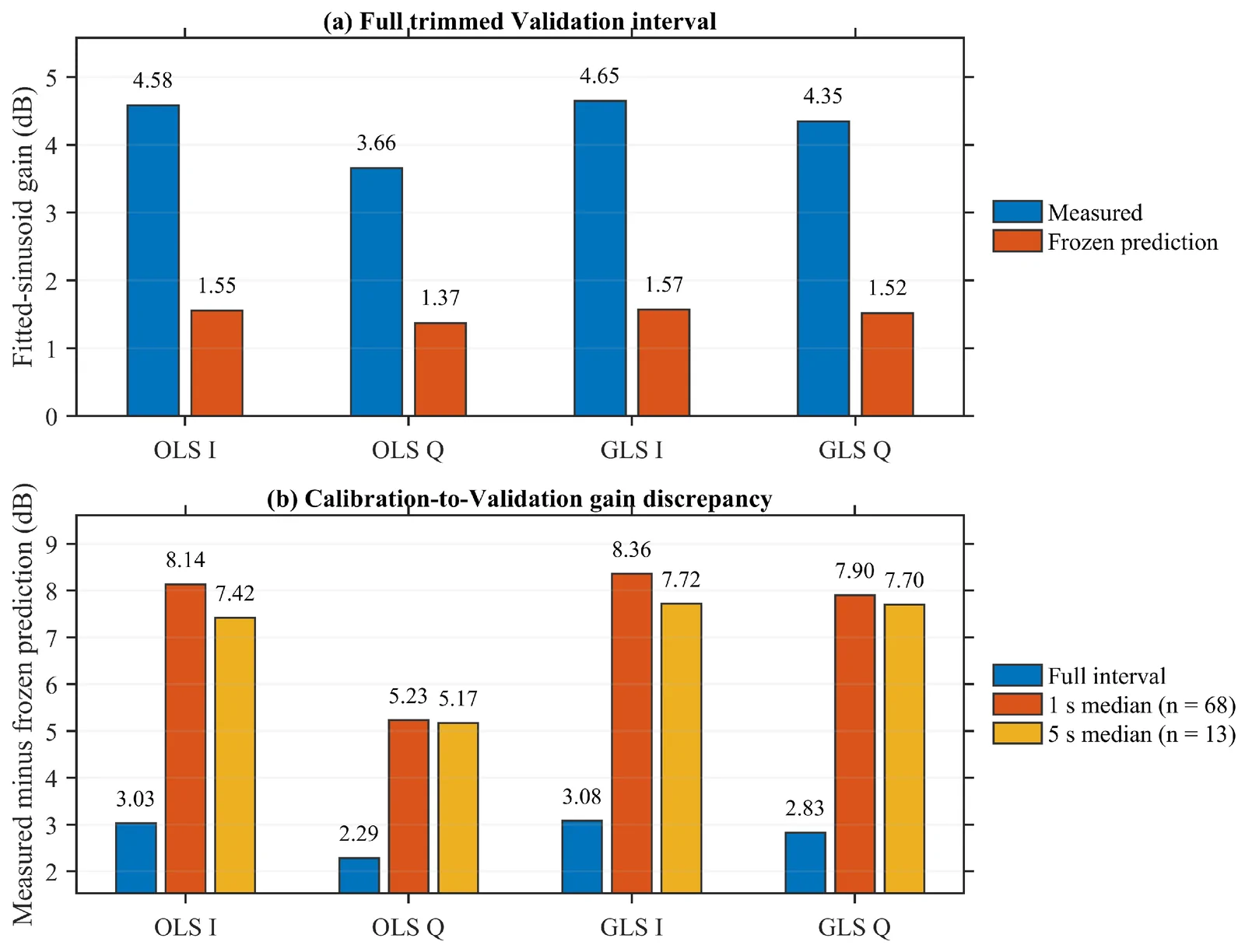
Calibration-to-Validation covariance-transfer diagnostics. (**a**) Measured and Calibration-covariance-predicted fitted-sinusoid gains over trimmed Validation. (**b**) Measured-minus-predicted discrepancies over the full interval and medians of the per-window discrepancies over 68 nonoverlapping 1 s windows and 13 nonoverlapping 5 s windows. These fitted-sinusoid results are distinct from the primary spectral-SNR metric.

Across 68 nonoverlapping 1 s windows, the median discrepancies were 8.136, 5.231, 8.358, and 7.902 dB; across 13 nonoverlapping 5 s windows, they were 7.422, 5.174, 7.719, and 7.701 dB. Covariance closure remained near machine precision. Thus, the discrepancy reflected incomplete transfer of the Calibration residual statistics rather than an algebraic error in the frozen fusion matrices. The corresponding Calibration-to-Validation covariance-transfer diagnostics are shown in Figure 3.

### 3.5. Innovation and Residual Diagnostics

The marginal NIS statistics in Table 2 were of the expected order, but the normalized innovations were strongly temporally correlated, as shown in Figure 2. Their lag-1 autocorrelations were approximately 0.954–0.959, and 96–98% of the first 1000 positive-lag autocorrelations exceeded the nominal pointwise white-noise bound. Ljung–Box tests rejected whiteness for both components and filters.

**Figure 4:**
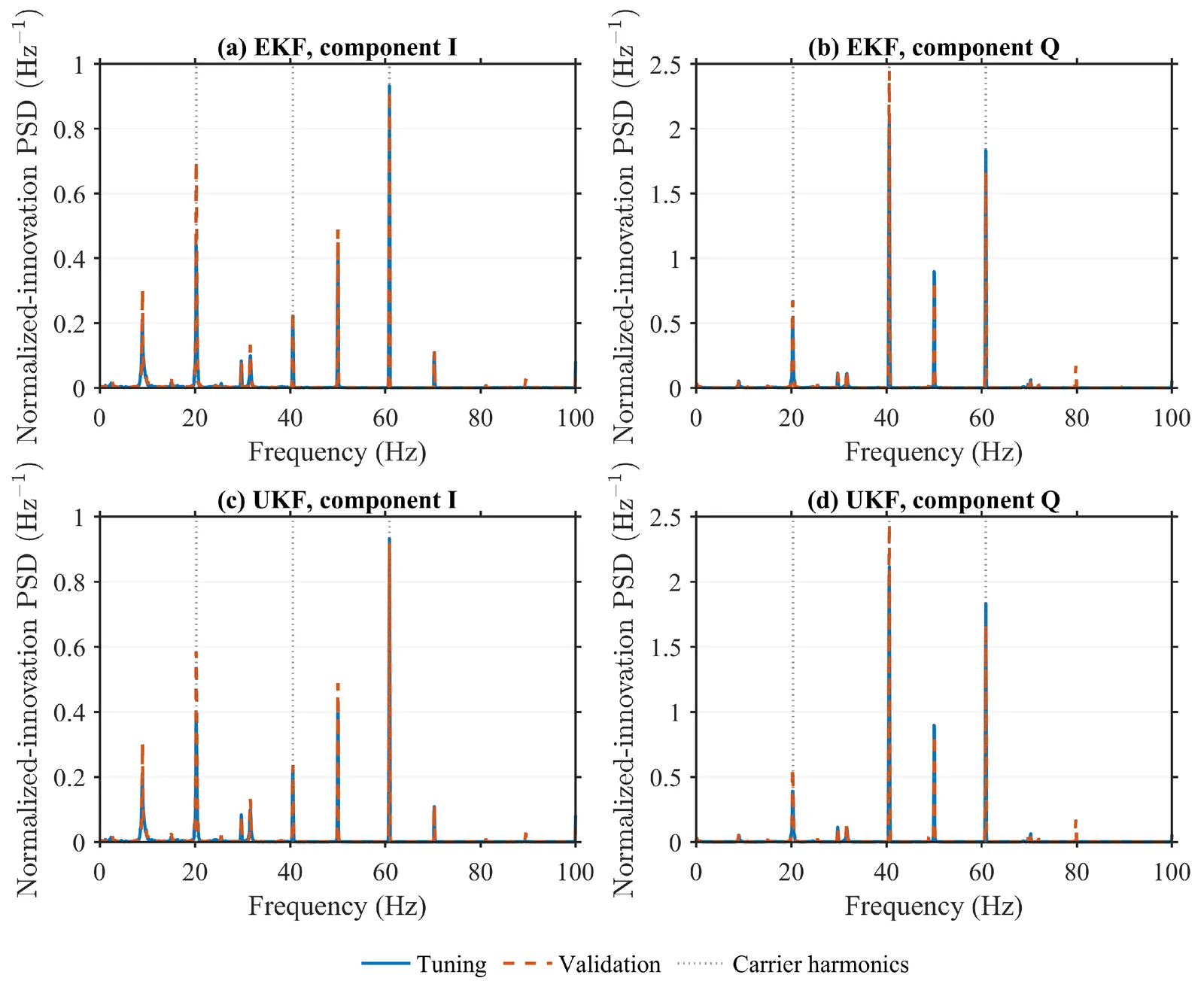
Welch spectra of the Cholesky-normalized innovations for the frozen integrated-process EKF and UKF. Panels show the I and Q innovation components over the steady Tuning segment and trimmed Validation. Welch spectra were computed using Hann-windowed segments with the same settings described in Appendix A.7.1. Vertical dotted lines denote the Calibration-frozen carrier frequency and its second and third harmonics. Coherent spectral structure persists across the two chronological segments, but this post hoc comparison was not used for formal estimator selection.

Welch spectra showed coherent structure near the 20.29 Hz carrier and its second and third harmonics. Across the EKF and UKF innovation components in Tuning and Validation, the detected peaks exceeded their local median spectral backgrounds by 20.87–35.08 dB, with magnitude-squared I/Q coherence values of 0.953–0.999. The spectra are shown in Figure 4; the complete carrier-harmonic table is retained in Appendix A.7.1.

A post hoc third-order phase-harmonic model fitted using Tuning data reduced substantial second- and third-harmonic content after transfer to Validation but did not reduce the maximum normalized autocorrelation. A subsequent VAR diagnostic reduced short-lag and cross-component correlation, but selected the maximum tested order, approached unit-root behavior, and still failed Validation whiteness tests. Because both analyses were motivated by inspection of the primary Validation residuals, they are reported only as Validation-informed diagnostics and were not incorporated into the formal estimators. Complete model definitions are provided in Appendix A.7.

The Tuning-fitted third-order phase-harmonic model reduced the transferred Validation second-harmonic peaks by approximately 19–22 dB and the third-harmonic peaks by approximately 24–31 dB. Depending on filter and component, the Validation phase-conditioned innovation RMS decreased by 57–83%. The carrier-frequency residual and the maximum normalized autocorrelation nevertheless persisted.

The subsequent VAR diagnostic selected the upper tested order, p=100, for both filters, with companion-matrix spectral radii near 0.9997. Lag-1 autocorrelation decreased from approximately 0.96 to about 0.05 for the I component and 0.20 for the Q component, and contemporaneous I/Q correlation decreased to approximately 0.01. The remaining Validation residuals still failed the Ljung–Box whiteness tests, so neither post hoc model was incorporated into the formal estimators.

## 4. Discussion

The held-out OLS I/Q spectral-SNR gains of 2.247 and 2.373 dB were below the ideal 3.0103-dB equal-noise reference, while GLS increased them to 2.450 and 2.814 dB. The full-rank, well-conditioned Calibration design matrix excludes rank deficiency as the explanation for the nonideal gain. Unequal gains and residual variances, interchannel correlation, segment-dependent statistics, and the operational spectral-SNR definition all contribute. The separate transfer diagnostic further showed that algebraically correct covariance propagation did not imply quantitative transfer of the Calibration residual model to Validation.

Previous ring-laser studies have applied Kalman filtering to compensate laser-parameter fluctuations and have emphasized the importance of nonlinear intracavity and laser dynamics in rotation-signal recovery [8,24,25]. The present framework addresses a complementary detector-side problem: joint gain–phase calibration of four nonideal readout channels, covariance-aware OLS/GLS fusion, and independently tuned recursive frequency estimation. Its Tuning-only sliding-window FFT reference is related to classical finite-record single-tone estimation [13], but it was used only as a common parameter-selection trend rather than as an absolute frequency truth. The strong experimental innovation correlation is consistent with covariance-model mismatch and with the limitations of marginal Kalman consistency tests [17,21]. Direct numerical comparison with earlier ring-laser studies is not appropriate because the present experiment used a different detector geometry and lacked an external rotation truth.

The known-truth dynamic tests showed that the predefined low-process settings, representative of the broad low-process plateaus selected for the nearly stationary experimental record, were not universal tracking settings: they minimized constant-frequency dispersion but produced substantial lag under prescribed dynamics, whereas the predefined Tracking configurations reduced transient error. Independent Tuning placed both filters on broad low-process-noise plateaus. The integrated phase–frequency covariance is theoretically more complete than the empirical diagonal model, but its additional terms were negligible at the selected settings. On this single Validation record, the EKF had lower dispersion and pseudo-reference-relative RMSE than the UKF. Because the reference was frozen from Tuning rather than obtained from an external standard, this comparison describes within-record repeatability rather than absolute frequency accuracy or general estimator superiority. From a computational perspective, the OLS and GLS fusion stages are fixed sample-wise linear transformations and do not introduce recursive smoothing delay. For the five-state recursive estimators, the EKF requires Jacobian evaluation at each update, whereas the UKF propagates 11 sigma points and therefore has a higher computational burden. Both estimators operate sample by sample after initialization. The sliding-window FFT reference was used only for Tuning and parameter selection and was not included in the final recursive estimation output path; therefore, the reported recursive frequency output does not contain additional FFT-window latency.

The experimental innovations exposed the main stochastic-model limitation. Near-reference marginal NIS values coexisted with autocorrelations near 0.96 and carrier-synchronous harmonic structure. Post hoc harmonic and VAR diagnostics reduced selected features but did not produce white residuals and were therefore excluded from the formal estimator. Possible causes include nonlinear I/Q geometry, harmonic distortion, multiplexed-channel timing, and unmodeled amplitude or bias dynamics; the present record cannot identify a unique mechanism.

This study is limited to one experimental record, lacks an external instantaneous-frequency truth, and does not preserve the multiplexed ADC channel timing or complete analog-front-end settings. The chronological split prevents direct within-record parameter leakage but does not establish cross-session generalization. Independent records with documented timing and analog response are required to test any prospectively specified colored or carrier-synchronous measurement model.

For reference, an independent long-duration single-channel frequency record from the same ring laser gyroscope was examined using Allan deviation analysis. This independent characterization indicated a random-walk coefficient of approximately $4.88\times 10^{-9}$ rad/s$/\sqrt{s}$ and a bias instability of approximately $2.22\times 10^{-9}$ rad/s at an averaging time of 20 s.

## 5. Conclusions

A gain–phase-calibrated multichannel I/Q fusion and recursive beat-frequency estimation framework has been developed and experimentally evaluated for a square He–Ne ring laser gyroscope. The proposed approach integrates calibration-based channel correction, covariance-aware fusion, and recursive frequency estimation within a unified processing chain.

Simulation and experimental results demonstrate that the proposed fusion strategy effectively improves the spectral quality of the multichannel I/Q signal, while the independently tuned recursive estimators achieve stable millihertz-level frequency estimation performance on held-out data.

The innovation analysis shows that covariance consistency and numerical stability alone are insufficient to fully characterize the stochastic behavior of practical ring-laser measurements, because carrier-synchronous temporal correlations remain in the residuals. These findings highlight the necessity of more comprehensive residual modeling and independent validation procedures for future high-precision frequency estimation systems.

## Figures and Tables

**Table 1:** Held-out Validation signal-to-noise ratios (SNRs) of the individual detector channels and ordinary least-squares (OLS) and generalized least-squares (GLS) fused components. Gains are referenced to the arithmetic mean of the four single-channel SNR values expressed in decibels.

Signal	SNR (dB)	Gain (dB)
Validation channel 1	36.854	−0.305
Validation channel 2	39.056	1.898
Validation channel 3	35.496	−1.662
Validation channel 4	37.228	0.069
Mean channel SNR in dB	37.159	0
OLS fused I	39.406	2.247
OLS fused Q	39.532	2.373
GLS fused I	39.609	2.450
GLS fused Q	39.973	2.814

**Table 2:** Frozen held-out Validation results for the independently tuned extended Kalman filter (EKF) and unscented Kalman filter (UKF) using the integrated process covariance. Reference-relative root-mean-square error (RMSE) metrics use the scalar pseudo-reference frozen from the Tuning-only sliding-window FFT trend. NIS denotes normalized innovation squared.

Metric	EKF	UKF
Mean frequency (Hz)	20.283478	20.283588
Reference-relative bias (mHz)	0.966	1.076
Reference-relative RMSE (mHz)	1.057	1.182
Frequency standard deviation (mHz)	0.429	0.490
Frequency peak-to-peak range (mHz)	1.571	1.812
Mean NIS	2.403	2.354
Median NIS	1.562	1.545
NIS 95% exceedance rate	8.82%	8.39%
NIS 99% exceedance rate	2.76%	2.52%

**Table 3:** Matched-model consistency results averaged over 100 paired Monte Carlo realizations. The theoretical means of the NIS, frequency-state NEES, and full-state NEES are 2, 1, and 5, respectively. Coverage denotes the fraction within the corresponding central 95% chi-square interval.

Statistic	Tracking EKF	Tracking UKF
Mean NIS	1.9988	1.9988
Central-95% NIS coverage	95.001%	95.001%
Mean frequency-state NEES	1.0163	1.0164
Central-95% frequency-state NEES coverage	94.740%	94.739%
Mean full-state NEES	5.0258	4.9900
Central-95% full-state NEES coverage	94.488%	94.553%

**Table 4:** Known-ground-truth frequency RMSEs for the four dynamic profiles. Each entry gives the Monte Carlo mean on the first line and the 95% confidence interval of the ensemble mean on the second line, all expressed in mHz.

Truth Profile	Low-Process EKF	Low-Process UKF	Tracking EKF	Tracking UKF
Constant	0.104 [0.095,0.112]	0.104 [0.095,0.113]	1.036 [1.027,1.045]	1.036 [1.027,1.045]
Step	67.491 [67.489,67.492]	62.019 [62.016,62.022]	7.842 [7.828,7.855]	7.842 [7.828,7.855]
Ramp	17.528 [17.527,17.529]	11.967 [11.967,11.968]	1.144 [1.135,1.153]	1.144 [1.135,1.153]
Sinusoidal	38.863 [38.862,38.864]	39.823 [39.822,39.824]	5.214 [5.211,5.218]	5.215 [5.211,5.219]

**Figure A1:**
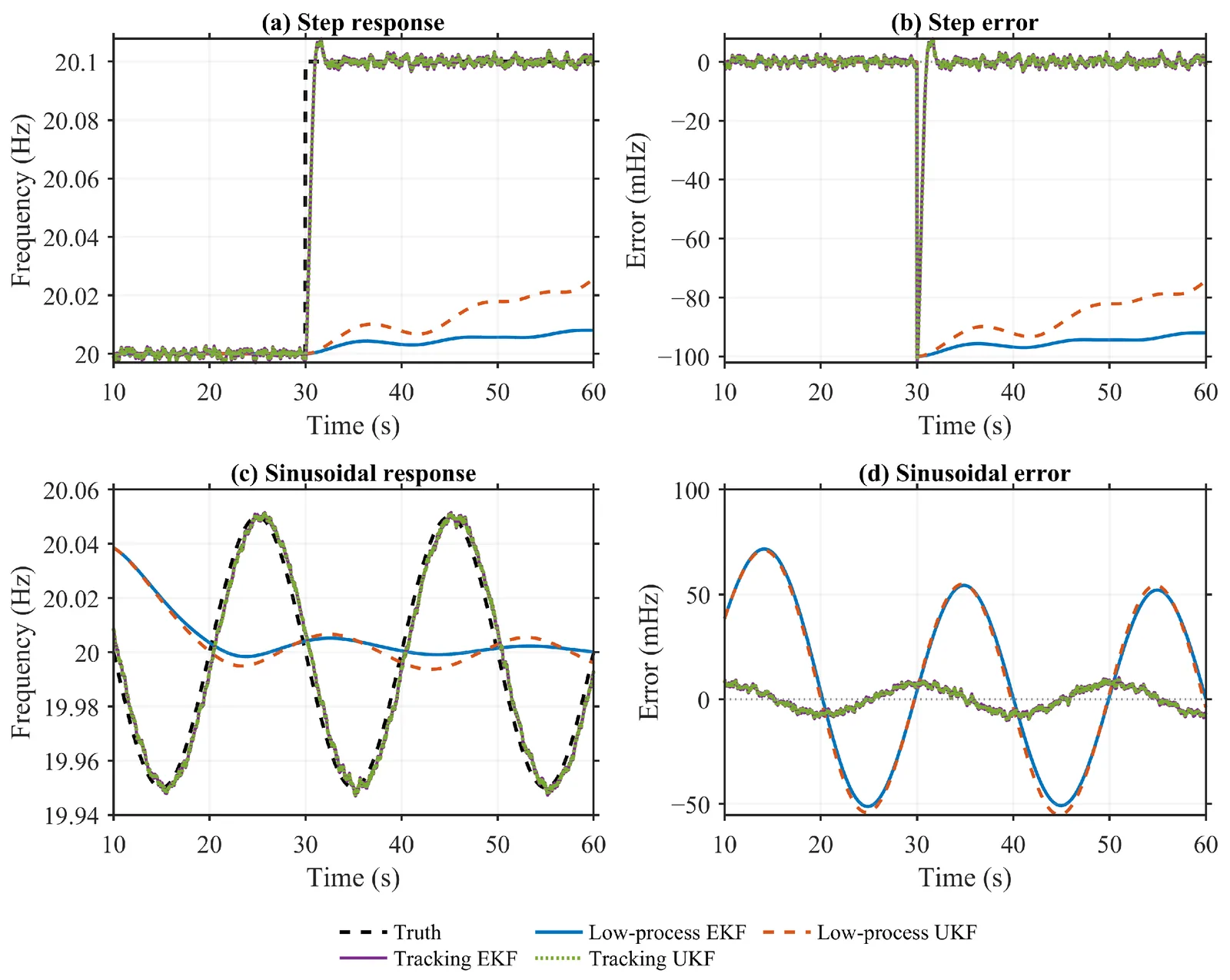
Representative known-ground-truth step and sinusoidal frequency-tracking results for the predefined low-process and Tracking configurations of both filters. (**a**) Frequency estimates for the step profile. (**b**) Corresponding step-tracking errors. (**c**) Frequency estimates for the sinusoidal profile. (**d**) Corresponding sinusoidal tracking errors. The displayed trajectories correspond to the first realizations in their respective predefined seed sequences and were not selected retrospectively according to tracking performance. The summary metrics in main-text Table 4 excluded the first and last 1 s of each realization and were averaged over 100 independent Monte Carlo realizations.

## Data Availability

The raw data supporting the conclusions of this article will be made available by the authors on request.

## Abbreviations

The following abbreviations are used in this manuscript:

ACF	Autocorrelation function
APD	Avalanche photodiode
DAQ	Data acquisition
EKF	Extended Kalman filter
FFT	Fast Fourier transform
GLS	Generalized least squares
I/Q	In-phase/quadrature
NEES	Normalized estimation error squared
NIS	Normalized innovation squared
OLS	Ordinary least squares
PSD	Power spectral density
RMSE	Root-mean-square error
SNR	Signal-to-noise ratio
UKF	Unscented Kalman filter
VAR	Vector autoregressive

## Appendix A. Detailed Methods and Supporting Analyses

This appendix retains implementation-level derivations, simulation matrices and seed mappings, numerical-audit details, and full diagnostic tables. The principal experimental and simulation findings are presented in the main text.

### Appendix A.1. Fusion and Ideal-Gain Derivations

#### Appendix A.1.1. Joint Gain–Phase Observation Model

After subtraction of the offsets estimated from Calibration, the four detector outputs were represented as

(A1) $$s_{n}=Hu_{n}+n_{n},\;u_{n}={\left[\begin{array}{cc} I_{n} & Q_{n} \end{array}\right]}^{T},$$

where

(A2) $$I_{n}=A_{n}\sin\theta_{n},\;Q_{n}=A_{n}\cos\theta_{n},$$

and the *k*th row of the Calibration-frozen design matrix was

(A3) $$H_{k,:}={\hat{g}}_{k,\mathrm{cal}}\left[\begin{array}{cc} \cos{\hat{\varphi }}_{k,\mathrm{cal}} & \sin{\hat{\varphi }}_{k,\mathrm{cal}} \end{array}\right].$$

The residual vector was assumed to have zero mean and covariance

(A4) $$E\left[n_{n}n_{n}^{T}\right]=\Sigma_{\mathrm{ch}}.$$

In the experimental analysis, ${\hat{\Sigma }}_{\mathrm{ch},\mathrm{cal}}$ was estimated from the four fixed-frequency Calibration regression residuals. It can therefore contain detector noise, waveform mismatch, and short-term signal variation and is interpreted as a residual covariance rather than a pure detector-noise covariance.

#### Appendix A.1.2. OLS and GLS Fusion

The ordinary least-squares estimate minimized

(A5) $${\hat{u}}_{\mathrm{OLS},n}=\arg\min_{u}{∥s_{n}-Hu∥}_{2}^{2}.$$

For full-column-rank H,

(A6) $${\hat{u}}_{\mathrm{OLS},n}=W_{\mathrm{OLS}}s_{n},\;W_{\mathrm{OLS}}={\left(H^{T}H\right)}^{-1}H^{T}.$$

Because $W_{\mathrm{OLS}}H=I_{2}$, the estimate is unbiased under the frozen linear model. Its propagated residual covariance is

(A7) $$R_{\mathrm{OLS}}=W_{\mathrm{OLS}}\Sigma_{\mathrm{ch}}W_{\mathrm{OLS}}^{T}.$$

When the channel residuals are correlated or have unequal variances, the generalized least-squares estimate minimizes

(A8) $${\hat{u}}_{\mathrm{GLS},n}=\arg\min_{u}{\left(s_{n}-Hu\right)}^{T}\Sigma_{\mathrm{ch}}^{-1}\left(s_{n}-Hu\right).$$

The corresponding fusion matrix is

(A9) $$W_{\mathrm{GLS}}={\left(H^{T}\Sigma_{\mathrm{ch}}^{-1}H\right)}^{-1}H^{T}\Sigma_{\mathrm{ch}}^{-1},$$

and its propagated residual covariance reduces to

(A10) $$R_{\mathrm{GLS}}={\left(H^{T}\Sigma_{\mathrm{ch}}^{-1}H\right)}^{-1}.$$

OLS was used as the primary recursive-filter input in the experimental analysis. GLS was evaluated as a covariance-aware fusion extension.

#### Appendix A.1.3. Ideal Equal-Noise Gain

For unit relative gains and ideal phase offsets,

(A11) $$\varphi_{\mathrm{ideal}}={\left[\begin{array}{cccc} 0 & \pi /4 & \pi /2 & 3\pi /4 \end{array}\right]}^{T},$$

and the design matrix satisfies

(A12) $$H^{T}H=2I_{2}.$$

For independent equal-variance channel residuals, $\Sigma_{\mathrm{ch}}=\sigma^{2}I_{4}$, the OLS output covariance is

(A13) $$R_{\mathrm{OLS}}=\sigma^{2}{\left(H^{T}H\right)}^{-1},$$

which, by Equation (A12), reduces to the $\frac{\sigma^{2}}{2}I_{2}$ result stated in Equation (6).

Each fused component therefore retains the signal amplitude, while its noise variance is reduced by a factor of two. The corresponding ideal gain relative to one input channel is

(A14) $$G_{\mathrm{ideal},\mathrm{dB}}=10\log_{10}\left(\frac{\sigma^{2}}{\sigma^{2}/2}\right)=10\log_{10}\left(2\right).$$

This evaluates to the 3.0103 dB equal-noise reference stated in Equation (6). This result is an equal-noise reference and is not a universal upper bound for the experimental unequal-noise and correlated-residual case.

### Appendix A.2. Recursive-Filter Implementation Details

#### Appendix A.2.1. State and Covariance Models

Both recursive estimators used the state vector

(A15) $$x_{k}={\left[\begin{array}{ccccc} \theta_{k} & \omega_{k} & A_{k} & b_{I,k} & b_{Q,k} \end{array}\right]}^{T},$$

where $\theta_{k}$ is phase, $\omega_{k}$ is angular frequency, $A_{k}$ is amplitude, and $b_{I,k}$ and $b_{Q,k}$ are the fused-component offsets. The deterministic transition and measurement functions were

(A16) $$f\left(x_{k}\right)=\left[\begin{array}{c} \theta_{k}+\omega_{k}T_{s}\\ \omega_{k}\\ A_{k}\\ b_{I,k}\\ b_{Q,k} \end{array}\right],\;h\left(x_{k}\right)=\left[\begin{array}{c} A_{k}\sin\theta_{k}+b_{I,k}\\ A_{k}\cos\theta_{k}+b_{Q,k} \end{array}\right].$$

The empirical process covariance was

(A17) $$Q_{\mathrm{emp}}=\mathrm{diag}\left[10^{-10},q_{\omega },10^{-10},10^{-12},10^{-12}\right],$$

where

(A18) $$q_{\omega }={\left(2\pi \sigma_{f,\mathrm{rw}}\right)}^{2}T_{s}.$$

The integrated phase–frequency covariance was

(A19) $$Q_{\mathrm{int}}=\left[\begin{array}{ccccc} 10^{-10}+q_{\omega }T_{s}^{2}/3 & q_{\omega }T_{s}/2 & 0 & 0 & 0\\ q_{\omega }T_{s}/2 & q_{\omega } & 0 & 0 & 0\\ 0 & 0 & 10^{-10} & 0 & 0\\ 0 & 0 & 0 & 10^{-12} & 0\\ 0 & 0 & 0 & 0 & 10^{-12} \end{array}\right].$$

The measurement covariance used during formal Tuning and Validation was

(A20) $$R=R_{\mathrm{scale}}W_{\mathrm{OLS}}{\hat{\Sigma }}_{\mathrm{ch},\mathrm{cal}}W_{\mathrm{OLS}}^{T}+10^{-12}I_{2}.$$

The four-channel residual covariance, OLS fusion matrix, and diagonal loading were frozen before Tuning. Neither filter re-estimated the measurement covariance from Validation.

#### Appendix A.2.2. Input-Dependent Initialization

The detector voltages were acquired at a nominal sampling rate of 1 kHz. The sampling frequency reconstructed from the archived time axis was represented in double precision by a value slightly below 1000 Hz. The initialization sample count was therefore

(A21) $$N_{0}=\min\;\left[N,\;\max\;\left(1,\left\lfloor f_{s}\right\rfloor \right)\right]=999,$$

where *N* is the number of samples in the current recursive run. The one-sample difference from the nominal 1 kHz count reflects the floating-point representation of the archived time axis rather than a physically meaningful sampling-rate deviation.

At the beginning of every Tuning or Validation run, the phase, amplitude, and I/Q offset states were initialized from the first $N_{0}$ fused samples of that run. The angular-frequency state was initialized using the frequency $f_{\mathrm{cal}}$ frozen from Calibration. The common nominal initialization settings of the EKF and UKF are summarized in Table A1.

The argument order in the phase initialization follows $I_{k}=A_{k}\sin\theta_{k}+b_{I,k}$ and $Q_{k}=A_{k}\cos\theta_{k}+b_{Q,k}$. The initial angular-frequency variance corresponds to a frequency standard deviation of 2 Hz.

If the median-radius amplitude estimate was nonfinite or nonpositive, both implementations first used

(A22) $${\hat{A}}_{0}=\sqrt{\mathrm{var}\left(I_{k}\right)+\mathrm{var}\left(Q_{k}\right)}.$$

The EKF additionally included a final unit-amplitude fallback if this quantity remained invalid, whereas the UKF did not include the second fallback. This difference concerned only the exceptional invalid-input path; the nominal initialization in Table A1 was common to both filters.

**Table A1:** Common nominal initialization of the experimental EKF and UKF. Here, $N_{0}=999$, and $f_{\mathrm{cal}}$ denotes the beat-frequency estimate frozen from Calibration.

Quantity	Definition	Data Source
${\hat{b}}_{I,0}$	$N_{0}^{-1}\sum_{k=1}^{N_{0}}I_{k}$	First $N_{0}$ fused I samples
${\hat{b}}_{Q,0}$	$N_{0}^{-1}\sum_{k=1}^{N_{0}}Q_{k}$	First $N_{0}$ fused Q samples
${\hat{\theta }}_{0}$	$\mathrm{atan}2\left(I_{1}-{\hat{b}}_{I,0},Q_{1}-{\hat{b}}_{Q,0}\right)$	First centered I/Q sample
${\hat{\omega }}_{0}$	$2\pi f_{\mathrm{cal}}$	Frozen Calibration estimate
${\hat{A}}_{0}$	${\mathrm{median}}_{1\leq k\leq N_{0}}\sqrt{{\left(I_{k}-{\hat{b}}_{I,0}\right)}^{2}+{\left(Q_{k}-{\hat{b}}_{Q,0}\right)}^{2}}$	First $N_{0}$ fused I/Q samples
$P_{0}$	$\mathrm{diag}\left[1,\;{\left(2\pi \times 2\right)}^{2},\;{\left(0.5{\hat{A}}_{0}\right)}^{2},\;0.5^{2},\;0.5^{2}\right]$	Frozen implementation

#### Appendix A.2.3. EKF Implementation

The EKF transition and measurement Jacobians were

(A23) $$F_{k}=\left[\begin{array}{ccccc} 1 & T_{s} & 0 & 0 & 0\\ 0 & 1 & 0 & 0 & 0\\ 0 & 0 & 1 & 0 & 0\\ 0 & 0 & 0 & 1 & 0\\ 0 & 0 & 0 & 0 & 1 \end{array}\right],$$

and

(A24) $$H_{k}^{\left(\mathrm{EKF}\right)}=\left[\begin{array}{ccccc} A_{k}\cos\theta_{k} & 0 & \sin\theta_{k} & 1 & 0\\ -A_{k}\sin\theta_{k} & 0 & \cos\theta_{k} & 0 & 1 \end{array}\right],$$

where the state-dependent quantities were evaluated at the predicted state. The posterior covariance used the Joseph stabilized form,

(A25) $$P_{k|k}=\left(I-K_{k}H_{k}^{\left(\mathrm{EKF}\right)}\right)P_{k|k-1}{\left(I-K_{k}H_{k}^{\left(\mathrm{EKF}\right)}\right)}^{T}+K_{k}RK_{k}^{T},$$

followed by explicit symmetrization.

#### Appendix A.2.4. UKF Implementation

The UKF used the scaled unscented transform with α=0.5, β=2, κ=0, and state dimension $n_{x}=5$. Thus,

(A26) $$\lambda =\alpha^{2}\left(n_{x}+\kappa \right)-n_{x},$$

and the sigma-point weights were

(A27) $$W_{0}^{\left(m\right)}=\frac{\lambda }{n_{x}+\lambda },\;W_{0}^{\left(c\right)}=W_{0}^{\left(m\right)}+1-\alpha^{2}+\beta ,$$

and

(A28) $$W_{i}^{\left(m\right)}=W_{i}^{\left(c\right)}=\frac{1}{2(n_{x}+\lambda )},\;i=1,\ldots ,2n_{x}.$$

The five-state filter propagated 11 sigma points. Phase means were calculated using circular averaging, and phase differences were wrapped to the principal interval before covariance accumulation. The posterior covariance was

(A29) $$P_{k|k}=P_{k|k-1}-K_{k}S_{k}K_{k}^{T},$$

followed by explicit symmetrization.

#### Appendix A.2.5. Constraints and Numerical Audit

The formal Tuning and Validation runs used an 18–22 Hz frequency interval. After each update, the phase was wrapped using atan2(sinθ,cosθ), negative amplitude estimates were reflected to the nonnegative half-line, and only the posterior frequency state was projected to the frozen interval. The posterior covariance was not recomputed after frequency projection.

The predicted state covariance, innovation covariance, and posterior state covariance were explicitly symmetrized. Adaptive stabilization was available before Cholesky factorization of the innovation covariance. The complete diagnostic mode recorded:

- Innovation-covariance Cholesky failures before adaptive regularization;
- Adaptive regularization events;
- Lower- and upper-frequency-bound activations;
- Minimum state-covariance eigenvalues;
- Minimum innovation-covariance eigenvalues.

The complete diagnostic mode was enabled for every candidate in all four Tuning scans and for the final held-out Validation runs.

### Appendix A.3. Detailed Simulation Configurations

#### Appendix A.3.1. Ideal Fusion Verification

The ideal verification used a single reproducible 100 s record sampled at 1 kHz, giving 100,000 samples. The beat frequency was 20 Hz, the I/Q amplitude was 1.5, and the initial phase was 0.37 rad. The four channels had unit relative gains and phase offsets of $0^{{}^{\circ}}$, $45^{{}^{\circ}}$, $90^{{}^{\circ}}$, and $135^{{}^{\circ}}$. Independent Gaussian noise with standard deviation 0.2 was added to each channel. MATLAB’s Mersenne Twister generator was initialized with seed 20250308.

The theoretical equal-noise OLS gain was the 3.0103 dB reference derived in Equation (A14).

The fixed-seed record yielded I/Q gains of 3.0178 and 3.0071 dB. Its maximum absolute noise-free reconstruction error was $2.22\times 10^{-16}$, and the covariance relative Frobenius error was 0.2391%.

#### Appendix A.3.2. Nonideal Covariance-Propagation Verification

The separate nonideal geometry used channel phases

(A30) $$\varphi_{\mathrm{sim}}={\left[\begin{array}{cccc} 0 & 46.9 & 91.1 & 134.9 \end{array}\right]}^{T}\deg,$$

static relative gains

(A31) $$g_{\mathrm{sim}}={\left[\begin{array}{cccc} 1.00 & 0.94 & 1.07 & 0.97 \end{array}\right]}^{T},$$

and channel-noise standard deviations

(A32) $$\sigma_{\mathrm{ch},\mathrm{sim}}={\left[\begin{array}{cccc} 0.080 & 0.095 & 0.075 & 0.090 \end{array}\right]}^{T}.$$

The channel-noise covariance was

(A33) $$\Sigma_{\mathrm{sim}}=\mathrm{diag}\left(\sigma_{\mathrm{ch},\mathrm{sim}}\right)C_{\mathrm{ch},\mathrm{sim}}\mathrm{diag}\left(\sigma_{\mathrm{ch},\mathrm{sim}}\right),$$

where

(A34) $$C_{\mathrm{ch},\mathrm{sim}}=\left[\begin{array}{cccc} 1.00 & 0.25 & 0.10 & -0.40\\ 0.25 & 1.00 & -0.12 & 0.20\\ 0.10 & -0.12 & 1.00 & 0.08\\ -0.40 & 0.20 & 0.08 & 1.00 \end{array}\right].$$

A total of 500,000 independent Gaussian residual vectors were generated with Mersenne Twister seed 21260300. The empirical and analytical output covariances were compared using

(A35) $$\epsilon_{R,m}=\frac{{∥{\hat{R}}_{m,\mathrm{MC}}-R_{m,\mathrm{pred}}∥}_{F}}{{∥R_{m,\mathrm{pred}}∥}_{F}},\;m\in \left\{\mathrm{OLS},\mathrm{GLS}\right\}.$$

The resulting errors were 0.160% for OLS and 0.139% for GLS.

#### Appendix A.3.3. Recursive-Filter Simulation Configuration and Matched Stochastic Experiment

The Stage-3 recursive-filter simulations used the nominal filter state and covariance

(A36) $$\begin{array}{cc} {\hat{x}}_{0} & ={\left[\begin{array}{ccccc} 0 & 2\pi f_{0,\mathrm{sim}} & 1 & 0 & 0 \end{array}\right]}^{T},\\ P_{0} & =\mathrm{diag}\left[\begin{array}{ccccc} 0.50^{2} & {\left(2\pi \sigma_{f,\mathrm{init}}\right)}^{2} & 0.20^{2} & 0.10^{2} & 0.10^{2} \end{array}\right]. \end{array}$$

where $f_{0,\mathrm{sim}}$ and $\sigma_{f,\mathrm{init}}$ define the initial beat frequency and its standard deviation in hertz, respectively. The corresponding second state and covariance element are represented in angular-frequency units through the 2π conversion.

The Stage-3 recursive-filter simulations used the same angular-frequency state convention as the experimental filter implementation. Specifically, the second state was represented as angular frequency ω in rad/s, with the phase propagation model written as $\theta_{k+1}=\theta_{k}+\omega_{k}T_{s}$. The simulation UKF used α=0.5, β=2, and κ=0, matching the scaled unscented transform parameters of the experimental UKF documented in Appendix A.2. These settings were applied to both the deterministic dynamic-response cases and the matched stochastic consistency case. The frequency-random-walk strength was specified by $\sigma_{f,\mathrm{rw}}=3\times 10^{-3}$ Hz $s^{-1/2}$ and converted to angular-frequency process noise using the same 2π scaling as the experimental formulation. The phase-process standard deviation was $10^{-5}$ per sample, and the amplitude and two bias random-walk standard deviations were $2\times 10^{-4}$ in their normalized units per square root second.

For the matched stochastic case, the initial truth state was drawn independently from $N({\hat{x}}_{0},P_{0})$ and was propagated using the same angular-frequency transition model and complete process covariance used by the Tracking EKF and UKF. The simulated four-channel residual vector satisfied

(A37) $$n_{k}∼N\left(0,\Sigma_{\mathrm{sim}}\right),$$

and the corresponding fused measurement residual was

(A38) $$v_{k}=W_{\mathrm{OLS}}n_{k},\;\mathrm{Cov}\left(v_{k}\right)=W_{\mathrm{OLS}}\Sigma_{\mathrm{sim}}W_{\mathrm{OLS}}^{T}=R_{\mathrm{OLS}}.$$

Thus, the truth-generation transition, initial-state distribution, process covariance, and fused measurement covariance matched those declared by the two Tracking filters. The low-process configurations were not evaluated in this case because their process covariance was deliberately mismatched to the truth model.

Each of the 100 paired realizations contained 60,000 samples over 60 s at 1 kHz. For realization index $i_{\mathrm{run}}=1,\ldots ,100$, the random seed was

(A39) $$s_{i_{\mathrm{run}}}^{\mathrm{matched}}=20310301+i_{\mathrm{run}},$$

giving seeds 20310302–20310401. Within each realization, the Tracking EKF and Tracking UKF used the same truth and simulated measurement record. The first and last 1 s were excluded from the consistency statistics.

Frequency-state and full-state NEES were defined as

(A40) $$\epsilon_{\omega ,k}=\frac{{\left({\hat{\omega }}_{k}-\omega_{k}^{\mathrm{true}}\right)}^{2}}{{\left[P_{k}\right]}_{\omega \omega }},\;\epsilon_{x,k}={\left({\hat{x}}_{k}-x_{k}^{\mathrm{true}}\right)}^{T}P_{k}^{-1}\left({\hat{x}}_{k}-x_{k}^{\mathrm{true}}\right).$$

The phase component of the state error used the shortest signed angular difference.

The one-sided 95% NIS-threshold exceedance rate was 4.991% for both filters, close to its nominal 5% reference. These results verify the NIS and NEES calculations under a correctly specified model; they do not establish adequacy of the experimental measurement model.

### Appendix A.4. Dynamic-Response Simulation Details

#### Appendix A.4.1. Simulation Design

These dynamic-response Monte Carlo experiments were separate from the single fixed-seed ideal fusion verification reported in the main text.

Unless stated otherwise, the dynamic-response simulations used the nonideal four-channel gain–phase geometry, channel-noise covariance, fixed simulation initialization, and UKF scaling parameters documented in Appendix A.3. These standalone simulations used the same angular-frequency state convention as the experimental recursive-filter implementation described in Appendix A.2. Across the predefined simulation configurations, the frequency-process setting and deterministic truth profile were varied as described below, while the remaining simulation parameters were held fixed.

The deterministic stress cases included a common amplitude modulation of depth 0.08 at 0.015 Hz and slowly varying I/Q biases with peak amplitudes 0.035 and 0.030 at 0.010 Hz.

Four deterministic frequency profiles were evaluated:

(A41) $$\begin{array}{cc} f_{A}\left(t\right) & =20,\\ f_{B}\left(t\right) & =\left\{\begin{array}{cc} 20.0, & t<30\;s,\\ 20.1, & t\geq 30\;s, \end{array}\right.\\ f_{C}\left(t\right) & =20+10^{-3}t,\\ f_{D}\left(t\right) & =20+0.05\sin(2\pi \;0.05t), \end{array}\;\left[\mathrm{Hz}\right].$$

The profiles were defined in hertz for readability, while the recursive filters internally propagated the corresponding angular-frequency state ω(t)=2πf(t).

These cases assessed steady-state dispersion, step response, ramp tracking, and sinusoidal amplitude and phase response, respectively. Two predefined frequency-process settings were compared: $\sigma_{f,\mathrm{rw}}=10^{-15}\;\mathrm{Hz}\;s^{-1/2}$ for the low-process configuration and $3\times 10^{-3}\;\mathrm{Hz}\;s^{-1/2}$ for the Tracking configuration. These parameters were specified in frequency units and converted to the angular-frequency process noise used by the recursive filters through the same 2π relationship described in Appendix A.3.

Each profile was evaluated using 100 independent Monte Carlo realizations for the low-process and Tracking configurations of both the EKF and UKF. Each realization contained 60,000 samples over 60 s at a sampling frequency of 1 kHz. The dynamic-response study comprised 400 independently generated measurement records: four truth profiles with 100 Monte Carlo realizations per profile. Each record was processed using four predefined estimator configurations—the low-process and Tracking settings of both the EKF and UKF—giving 1600 filter executions in total. The archived base random seed was $s_{\mathrm{base}}=20260301$. For dynamic-profile index $i_{\mathrm{case}}=1,\ldots ,4$ and Monte Carlo realization index $i_{\mathrm{run}}=1,\ldots ,100$, MATLAB’s Mersenne Twister pseudorandom-number generator was initialized using

(A42) $$s_{i_{\mathrm{case}},i_{\mathrm{run}}}=s_{\mathrm{base}}+10000\;i_{\mathrm{case}}+i_{\mathrm{run}}.$$

The profile indices $i_{\mathrm{case}}=1,2,3,4$ corresponded to the constant-frequency, step, ramp, and sinusoidal cases, respectively. Consequently, each profile–realization pair used a distinct and reproducible Gaussian-noise sequence. Both filters and both predefined process-noise configurations within a given profile and realization were evaluated using the same simulated measurement record, ensuring a paired comparison. The first and last 1 s were excluded from the global bias, standard deviation, RMSE, absolute-error, and peak-to-peak summaries. Step crossing and settling metrics were evaluated relative to the prescribed step time using the definitions given below and were not calculated from the globally trimmed sequence alone.

Frequency errors were evaluated using bias, standard deviation, RMSE, mean and maximum absolute error, and peak-to-peak error. The step metrics used a 0.1 s moving average and conventional 10%, 50%, and 90% crossings. Settling required the estimate to remain within the larger of 2% of the step amplitude and three pre-step standard deviations for 1 s. Sinusoidal amplitude ratios and phase delays were obtained from harmonic fits at the prescribed modulation frequency.

For each truth profile and estimator configuration, the reported RMSE was averaged over the $N_{\mathrm{valid}}=100$ valid realizations. The 95% confidence interval of the ensemble mean was calculated using the normal approximation,

(A43) $$\bar{\mathrm{RMSE}}\pm 1.96\frac{s_{\mathrm{RMSE}}}{\sqrt{N_{\mathrm{valid}}}},$$

where $s_{\mathrm{RMSE}}$ is the sample standard deviation of the per-realization RMSE values. This interval characterizes uncertainty in the ensemble mean and is not a prediction interval for an individual realization or a pointwise tracking-error bound.

#### Appendix A.4.2. Dynamic Frequency-Tracking Results

The representative step and sinusoidal realizations are shown in Figure A1. The low-process setting exhibited substantial dynamic lag, whereas the Tracking EKF and UKF reduced the transient and sinusoidal errors. For the sinusoidal truth, the low-process EKF and UKF recovered modulation amplitude ratios of approximately 0.121 and 0.159, with phase delays of approximately 4.56 and 5.23 s, respectively. The Tracking EKF and UKF preserved approximately unity modulation amplitude and reduced the phase delay to approximately 0.468 s.

The displayed trajectories are the first realizations in their respective predefined seed sequences and were not selected according to tracking performance. The tabulated metrics are averages over 100 realizations per truth profile. The Tracking setting, $\sigma_{f,\mathrm{rw}}=3\times 10^{-3}$, was fixed across the truth profiles rather than optimized separately for each case.

The confidence intervals in main-text Table 4 quantify uncertainty in the ensemble mean RMSE and are not pointwise bounds for an individual trajectory. Their narrow widths primarily reflect deterministic tracking mismatch with a smaller contribution from between-realization measurement noise.

### Appendix A.5. Independent Tuning and Numerical Audit

#### Appendix A.5.1. Filter-Independent Tuning Reference

A common Tuning-only frequency reference was formed from the four offset-corrected detector channels. Each channel used a 300-sample symmetric Hann window, 10-sample step, 16,384-point Fourier transform, and 15–25 Hz peak-search band. Parabolic interpolation refined the dominant bin. The resulting 5971 estimates per channel were combined using nonnegative minimum-variance covariance weights estimated from the Tuning frequency matrix.

The window estimates were interpolated onto the sample-level Tuning time axis. Linear extrapolation was retained outside the first and last window centers to reproduce the predefined selection criterion. After the 1 s settling exclusion, 149 evaluated samples lay outside direct FFT-center support. The full-evaluation and support-only trend means differed by approximately 0.020 mHz.

#### Appendix A.5.2. Search Grid and Composite Cost

Four independent tasks were evaluated: EKF and UKF with the empirical and integrated process covariances. Each task scanned 40 logarithmically spaced values of $\sigma_{f,\mathrm{rw}}\in \left[10^{-15},10^{-2}\right]$ and 20 values of $R_{\mathrm{scale}}\in \left[10^{-3},10^{2}\right]$, giving 800 candidates per task and 3200 candidates in total.

The predefined composite cost was

(A44) $$\begin{array}{cc} J= & 0.40\left|\log\frac{\bar{\epsilon }}{2}\right|+0.25\left|\log\frac{\tilde{\epsilon }}{2\ln2}\right|\\ \; & +0.15\frac{\sigma_{\hat{f}}}{\mathrm{med}(\sigma_{\hat{f}})}+0.10\frac{P2P}{\mathrm{med}(P2P)}+0.10\frac{{\mathrm{RMSE}}_{\mathrm{trend}}}{\mathrm{med}({\mathrm{RMSE}}_{\mathrm{trend}})}. \end{array}$$

Here, $\bar{\epsilon }$ and $\tilde{\epsilon }$ are the mean and median NIS. The remaining terms measure frequency standard deviation, peak-to-peak range, and RMSE relative to the common Tuning-only trend. Normalization medians were calculated separately within each task, so absolute cost values were not compared across filters or process models.

Candidates were preferentially selected from

(A45) $$1.5\leq \bar{\epsilon }\leq 2.5,\;1.1\leq \tilde{\epsilon }\leq 1.8.$$

All four formal Tuning tasks contained valid candidates in the strict region. Consequently, the predefined wider-region and global minimum-cost fallback branches were not invoked and did not affect any reported parameter selection. Numerical validity additionally required positive state and innovation covariances, no innovation-covariance Cholesky failure, no activation of adaptive innovation-covariance regularization, and a frequency-bound-hit rate no greater than 1%.

#### Appendix A.5.3. Selected Parameters

At the selected $R_{\mathrm{scale}}$, the EKF 1%-near-optimal region extended approximately from $10^{-15}$ to $4.64\times 10^{-7}$ Hz $s^{-1/2}$, whereas the UKF region extended from $10^{-15}$ to $10^{-6}$ Hz $s^{-1/2}$. The selected process strengths are therefore numerical representatives of broad low-process-noise plateaus rather than precise physical estimates. The parameters selected independently for the four filter and process-covariance configurations are summarized in Table A2.

**Table A2:** Parameters selected independently using only the Tuning segment for the four EKF/UKF and process-covariance configurations.

Filter	Q Model	$\sigma_{f,\mathrm{rw}}$ $\left(\mathrm{Hz}\;s^{-1/2}\right)$	$R_{\mathrm{scale}}$	Mean NIS	Median NIS
EKF	Empirical	$1.0000\times 10^{-11}$	0.0695	2.1552	1.4495
EKF	Integrated	$1.0000\times 10^{-11}$	0.0695	2.1552	1.4495
UKF	Empirical	$4.6416\times 10^{-12}$	0.0695	2.1285	1.4275
UKF	Integrated	$4.6416\times 10^{-12}$	0.0695	2.1285	1.4275

The empirical and integrated models produced identical selected metrics at stored precision because the additional integrated phase–frequency covariance terms were negligible at the selected settings.

#### Appendix A.5.4. Numerical Audit

All four 800-candidate scans completed without an innovation-covariance Cholesky failure or activation of the adaptive regularization fallback. Each selected candidate belonged to the strict marginal-NIS region. The full audit also recorded covariance eigenvalues, frequency-bound activations, and nonfinite states. All selected parameters were frozen before Validation, and no Validation diagnostic was fed back into parameter selection.

### Appendix A.6. Calibration-to-Validation Covariance-Transfer Definition

A fitted-sinusoid diagnostic was used to test whether the channel residual covariance frozen from Calibration transferred quantitatively to held-out Validation. This diagnostic was separate from the primary spectral-SNR metric.

For a waveform fitted as

(A46) $$x\left(t\right)=a_{x}\sin\left(2\pi f_{\mathrm{fit}}t\right)+c_{x}\cos\left(2\pi f_{\mathrm{fit}}t\right)+d_{x}+r_{x}\left(t\right),$$

the fitted signal and residual powers were

(A47) $$P_{s,x}=\frac{a_{x}^{2}+c_{x}^{2}}{2},\;P_{r,x}=\frac{1}{N}\sum_{n=1}^{N}r_{x}^{2}\left(t_{n}\right),$$

and

(A48) $${\mathrm{SNR}}_{x,\mathrm{fit}}=10\log_{10}\left(\frac{P_{s,x}}{P_{r,x}}\right).$$

For fusion method m∈{OLS,GLS} and component q∈{I,Q}, the measured gain was

(A49) $$G_{m,q}^{\mathrm{meas}}={\mathrm{SNR}}_{m,q,\mathrm{fit}}-\frac{1}{4}\sum_{k=1}^{4}{\mathrm{SNR}}_{k,\mathrm{fit}}.$$

The Calibration covariance was propagated as

(A50) $$R_{m,\mathrm{cal}}=W_{m}{\hat{\Sigma }}_{\mathrm{ch},\mathrm{cal}}W_{m}^{T}.$$

The predicted channel and fused-component SNRs were

(A51) $${\mathrm{SNR}}_{k,\mathrm{fit}}^{\mathrm{pred}}=10\log_{10}\left[\frac{P_{s,k}}{{\left[{\hat{\Sigma }}_{\mathrm{ch},\mathrm{cal}}\right]}_{kk}}\right],$$

and

(A52) $${\mathrm{SNR}}_{m,q,\mathrm{fit}}^{\mathrm{pred}}=10\log_{10}\left[\frac{P_{s,m,q}}{{\left[R_{m,\mathrm{cal}}\right]}_{qq}}\right].$$

The predicted gain and discrepancy were

(A53) $$G_{m,q}^{\mathrm{pred}}={\mathrm{SNR}}_{m,q,\mathrm{fit}}^{\mathrm{pred}}-\frac{1}{4}\sum_{k=1}^{4}{\mathrm{SNR}}_{k,\mathrm{fit}}^{\mathrm{pred}},\;\Delta G_{m,q}=G_{m,q}^{\mathrm{meas}}-G_{m,q}^{\mathrm{pred}}.$$

Because the prediction used signal powers fitted from the evaluated Validation interval while retaining the covariance frozen from Calibration, it was a conditional covariance-transfer diagnostic rather than a fully prospective SNR prediction.

The corresponding held-out results are reported in Section 3.4 and Figure 3.

### Appendix A.7. Residual Diagnostic Definitions and Full Spectral Results

#### Appendix A.7.1. Innovation Spectral Diagnostics

The following analyses were performed after inspection of the primary held-out Validation innovations. They were therefore classified as Validation-informed diagnostics and were not used to select or modify any formal EKF or UKF parameter.

The frozen integrated-process EKF and UKF were rerun over Tuning using the parameters selected without Validation. The first 999 Tuning samples were excluded from the steady-state diagnostic. For both the remaining Tuning samples and the trimmed Validation samples, the two-component innovations were normalized by the Cholesky factor of their predicted innovation covariance.

The normalized-innovation spectra were estimated using Welch’s method with 8192-sample Hann segments, 4096-sample overlap, and a 16,384-point Fourier transform. Each segment was mean removed before windowing. One-sided auto- and cross-spectral estimates were averaged over all complete segments. Magnitude-squared I/Q coherence was calculated from the averaged auto- and cross-spectral densities.

Carrier-harmonic peaks were evaluated in ±1 Hz neighborhoods around the Calibration-frozen carrier frequency and its second and third harmonics. The local spectral background was defined as the median power within ±2 Hz of each target, excluding the central ±0.25 Hz interval. The peak-above-background level was calculated as the detected peak power spectral density (PSD) in decibels per hertz minus the corresponding local median background in decibels per hertz. Magnitude-squared I/Q coherence was evaluated at the common detected peak frequency. The resulting carrier-harmonic peak and coherence metrics are summarized in Table A3.

**Table A3:** Carrier-harmonic peaks in the Cholesky-normalized innovation spectra. The I and Q excess levels are the detected peak PSD values relative to the corresponding local median spectral backgrounds. The magnitude-squared coherence was evaluated between the I and Q innovations at the common detected peak frequency.

Segment	Order	Target (Hz)	Peak (Hz)	I Excess (dB)	Q Excess (dB)	Coherence
*EKF*						
Tuning	1	20.2866	20.2637	21.99	21.64	0.992
Tuning	2	40.5732	40.5884	23.43	32.80	0.993
Tuning	3	60.8598	60.8521	33.36	35.08	0.999
Validation	1	20.2866	20.2637	23.39	22.83	0.963
Validation	2	40.5732	40.5884	24.20	34.43	0.994
Validation	3	60.8598	60.8521	33.58	33.08	0.999
*UKF*						
Tuning	1	20.2866	20.2637	21.29	20.87	0.990
Tuning	2	40.5732	40.5884	23.43	32.80	0.993
Tuning	3	60.8598	60.8521	33.36	35.08	0.999
Validation	1	20.2866	20.2637	22.60	22.09	0.953
Validation	2	40.5732	40.5884	24.20	34.43	0.994
Validation	3	60.8598	60.8521	33.58	33.08	0.999

As summarized in Table A3, peaks occurred near the Calibration-frozen carrier and its second and third harmonics for both filters and chronological segments. Across all reported filter, segment, component, and harmonic combinations, the detected peaks exceeded their local median spectral backgrounds by 20.87–35.08 dB. The corresponding I/Q magnitude-squared coherence ranged from 0.953 to 0.999. The similar peak locations in Tuning and Validation indicate a persistent within-record carrier-synchronous component not represented by the formal sinusoidal measurement model. Because both segments were obtained from the same experimental record, this persistence does not establish cross-record generalization.

#### Appendix A.7.2. Tuning-Fitted Phase-Harmonic Diagnostic

After inspection of the primary Validation innovations revealed carrier-synchronous structure, a post hoc transfer diagnostic was defined. Its coefficients were fitted using only a chronological fit/check partition of Tuning and were not estimated from Validation.

For normalized innovation vector $\eta_{k}$ and frozen filter phase ${\hat{\theta }}_{k}$, the conditional-mean model was

(A54) $$\hat{\eta }\left({\hat{\theta }}_{k}\right)=b_{0}+\sum_{m=1}^{M}\left[b_{c,m}\cos\left(m{\hat{\theta }}_{k}\right)+b_{s,m}\sin\left(m{\hat{\theta }}_{k}\right)\right].$$

A chronological Tuning fit/check split selected M=3 for both filters. The coefficients were subsequently refitted using all steady Tuning samples, frozen, and transferred without refitting to Validation.

The transferred Validation effects are summarized in Section 3.5.

#### Appendix A.7.3. Validation-Informed VAR Diagnostic

A subsequent exploratory vector autoregressive model was fitted to the Tuning harmonic residuals,

(A55) $$e_{k}=\sum_{\ell =1}^{p}\Phi_{\ell }e_{k-\ell }+w_{k}.$$

The candidate orders were

(A56) p∈{0,…,10,15,20,30,40,50,60,75,100}.

The selected order and held-out residual effects are summarized in Section 3.5.
